# Supplementary material for: Citrullinated and Malondialdehyde–Acetaldehyde-Modified Fibrinogen Activates Macrophages and Promotes Coronary Endothelial Cell Inflammation
Source: Curr Issues Mol Biol. 2025 Nov 13;47(11):943. doi: 10.3390/cimb47110943 (PMC12651101; doi:10.3390/cimb47110943)
Supplement: Supplementary file 1 [file cimb-47-00943-s001.zip › cimb-3968155-supplementary.pdf]

Citrullinated and Malondialdehyde–Acetaldehyde-Modified Fibrinogen Activates Macrophages and Promotes Coronary Endothelial Cell Inflammation

Wenxian Zhou [1], Hannah J. Johnson [1], Michael J. Duryee [1,2], Nozima Aripova [1], Engle E. Sharp [1], Carlos D. Hunter [1,2], Kimberley Sinanan [1], Henry C. Drvol [1], Mason G. Feely [1], Tate M. Johnson [1,2], Mabruka Alfaidi [3], Daniel R. Anderson [4], Vineeth Reddy [4], Keshore Bidasee [5], Robert G. Bennett [2,6,7], Jill A. Poole [8], Geoffrey M. Thiele [1,2] and Ted R. Mikuls [1,2,*]

[1]Department of Internal Medicine, Division of Rheumatology, University of Nebraska Medical Center, Omaha, NE 68198, USA
[2]Veterans Affairs Nebraska-Western Iowa Health Care System, Omaha, NE 68105, USA
[3]Department of Cellular and Integrative Physiology, University of Nebraska Medical Center, Omaha, NE 68198, USA
[4]Department of Internal Medicine, Division of Cardiovascular Medicine, University of Nebraska Medical Center, Omaha, NE 68198, USA
[5]Department of Pharmacology & Experimental Neuroscience, University of Nebraska Medical Center, Omaha, NE 68198, USA
[6]Department of Internal Medicine, Division of Diabetes, Endocrinology and Metabolism, University of Nebraska Medical Center, Omaha, NE 68198, USA
[7]Department of Biochemistry and Molecular Biology, University of Nebraska Medical Center, Omaha, NE 68198, USA
[8]Department of Internal Medicine, Division of Allergy and Immunology, University of Nebraska Medical Center, Omaha, NE 68198, USA

***Correspondence:** tmikuls@unmc.edu; Tel.: +1-402-559-8168; Fax: +1-402-559-6788

Supplemental Material

Figure S1
Figure S2
Figure S3
Figure S4
Figure S5
Figure S6
Figure S7
Figure S8
Figure S9
Figure S10
Table S1
Table S2
Major Resources Table

Figure S1:

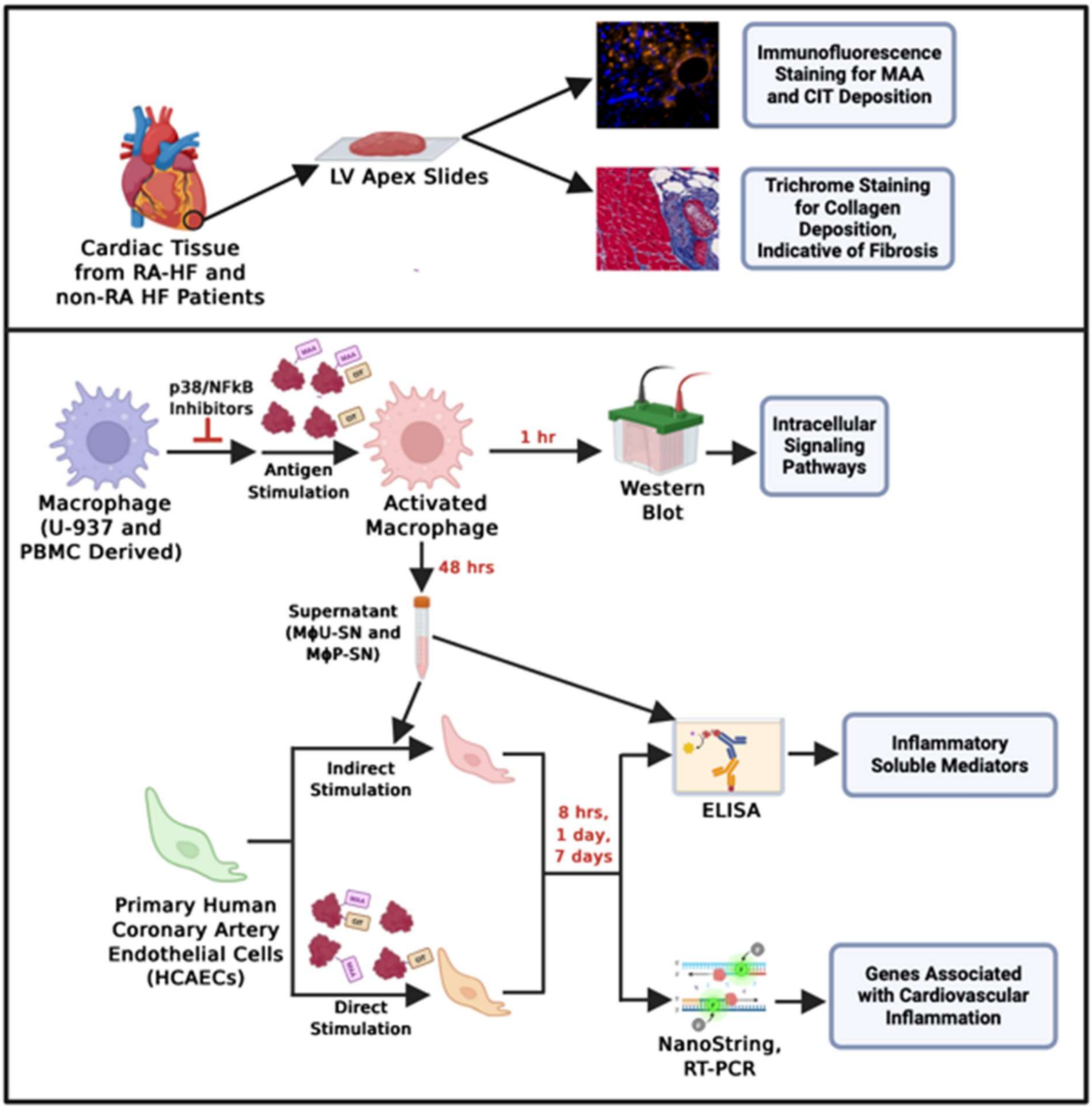

Study Schema. Left ventricular (LV) tissues from patients with rheumatoid arthritis (RA) and heart failure (HF) and non-RA HF controls (n=3 each) were assessed for the presence of malondialdehyde-acetaldehyde (MAA), citrullination (CIT), and collagen deposition consistent with fibrosis. In separate experiments, human macrophages were stimulated with unmodified fibrinogen (FIB), FIB-MAA, FIB-CIT, or FIB-MAA-CIT, in the presence or absence of NF-κB (BAY-11-7085) or p38 (BIRB-796) inhibitors. Macrophage intracellular signaling pathways were analyzed by Western blot and cytokine secretion measured by ELISA. Supernatants from macrophages stimulated with the different FIB antigens were applied to primary human coronary artery endothelial cells (HCAECs) to explore gene expression changes (by NanoString and RT-PCR) and soluble mediator secretion (by ELISA). PBMC = Peripheral Blood Mononuclear Cells; p38 = p38 mitogen-activated protein kinases; NF-κB = Nuclear Factor kappa B.

Figure S2:

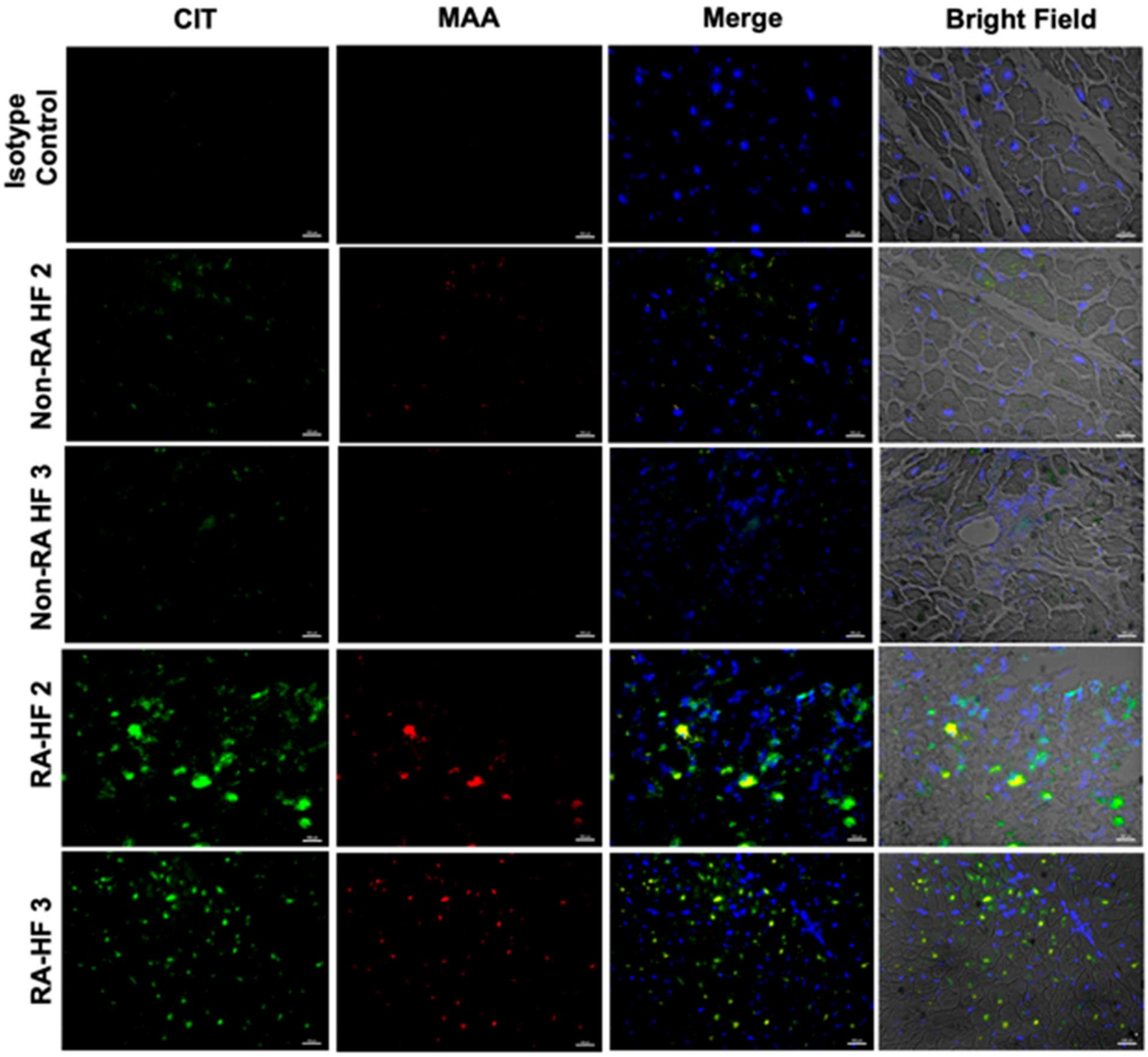

Immunofluorescence Images of citrullination (CIT) and malondialdehyde-acetaldehyde (MAA) in Human Cardiac Tissues. LV Apex Tissues from individuals with rheumatoid arthritis (RA) and heart failure (HF) and non-RA HF were stained for anti-mouse IgM isotype control (for CIT) and anti-rabbit IgG isotype control (for MAA). Images derived from tissues of 2 additional Non-RA HF and 2 additional RA-HF patients (not shown in **Fig. 1**). White scale bar = 100µm.

Figure S3:

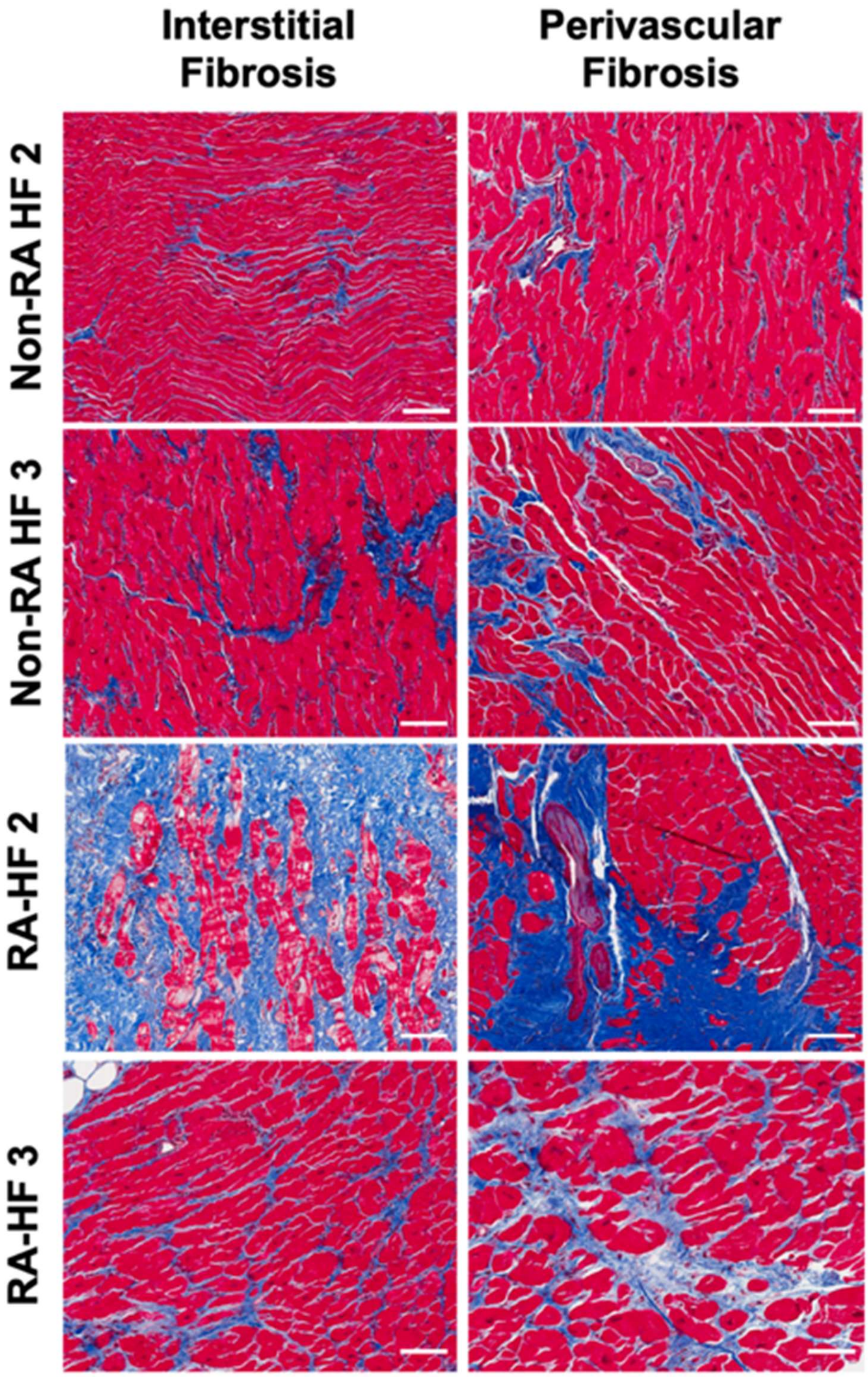

Interstitial and Perivascular Fibrosis in Human Myocardium. LV Apex Tissues from 2 additional individuals with rheumatoid arthritis (RA) and heart failure (HF) and 2 with Non-RA HF (not shown in **Fig. 1**) were stained with Trichrome (blue). White scale bar = 100µm.

Figure S4:

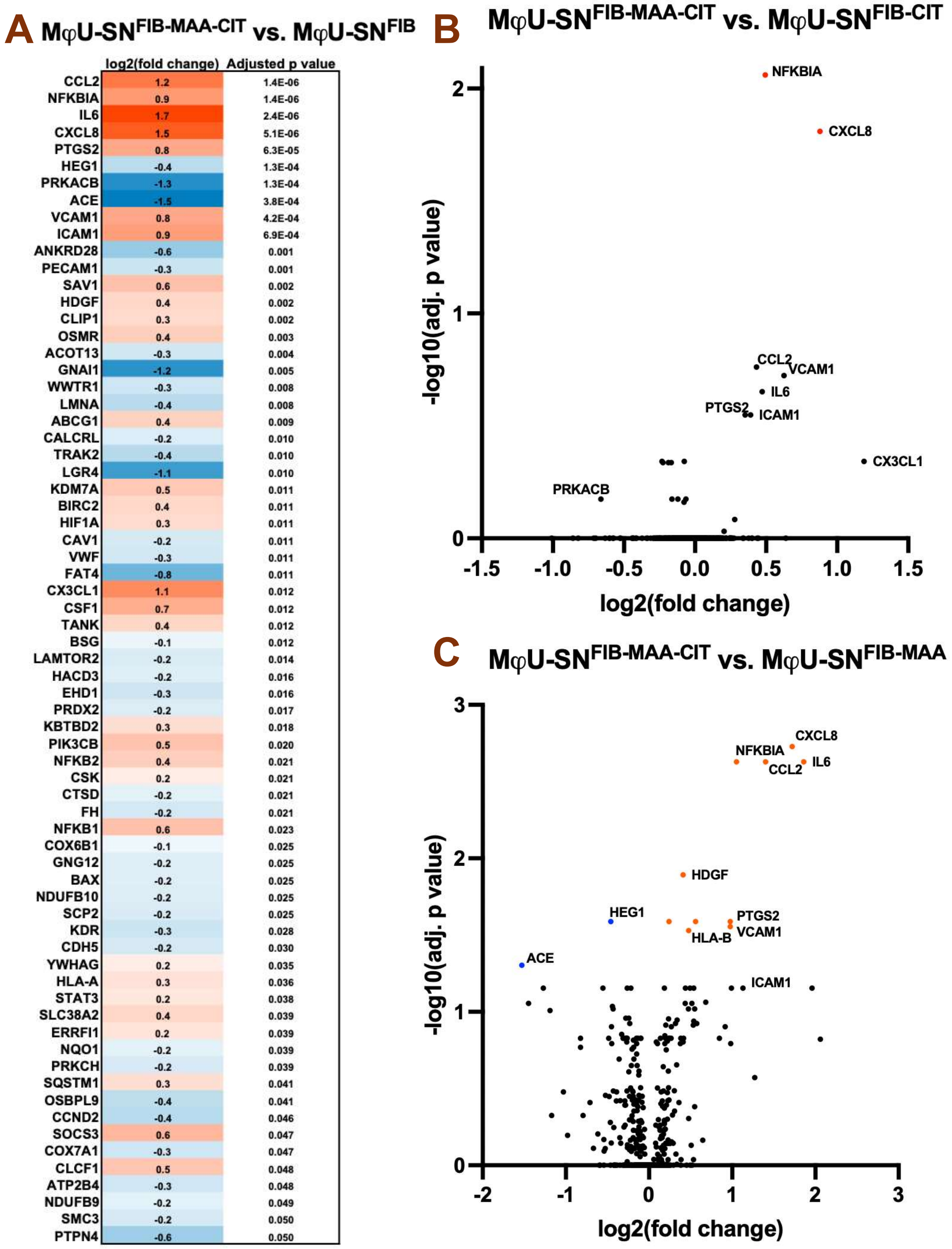

Human Coronary Artery Endothelial Cell (HCAEC) Differential Gene Expression after Treatment with Antigen-Stimulated Macrophage Supernatants. A) Full list of differentially expressed genes comparing HCAEC stimulated with MΦU-$SN^{FIB-MAA-CIT}$ to that of MΦU-SN^{FIB}. Orange color indicates upregulation, blue color indicates downregulation. **B-C)** Volcano plot comparing HCAEC stimulated with MΦU-$SN^{FIB-MAA-CIT}$ to that of **B)** MΦU-$SN^{FIB-CIT}$ and **C)** MΦU-$SN^{FIB-MAA}$. Colored dots indicate significant changes (adjusted p<0.05).

Figure S5:

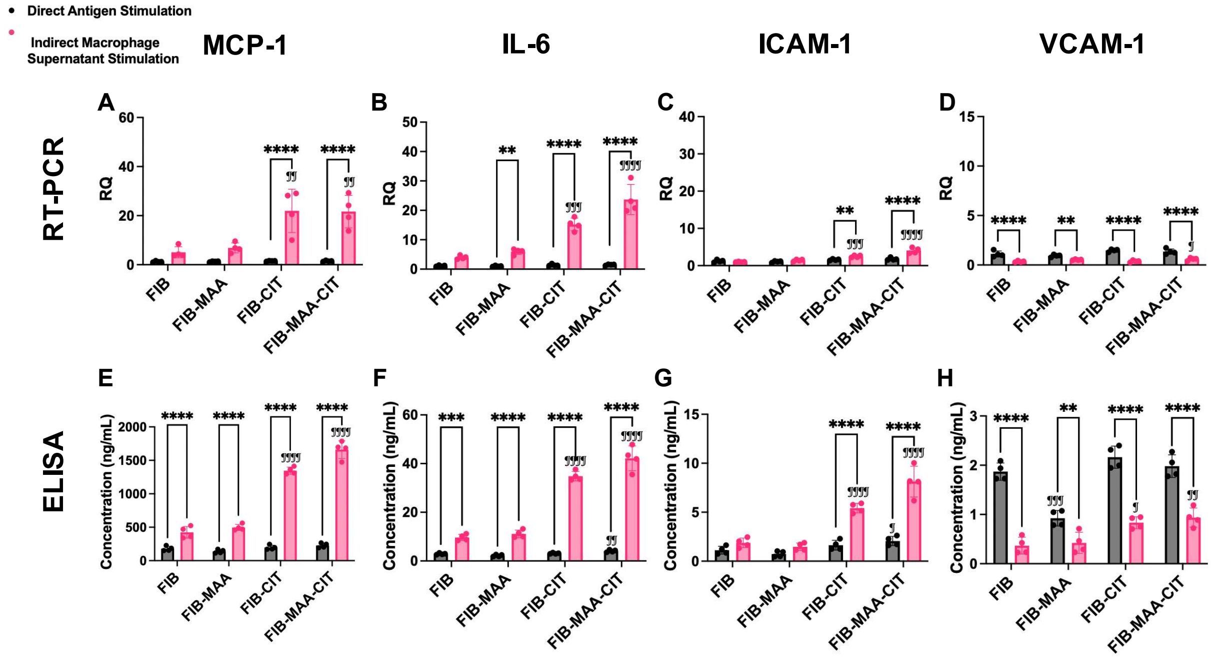

HCAEC Gene Expression Following 7-day Direct Antigen Stimulation and Indirect Stimulation with Macrophage Supernatants. MCP-1, IL-6, ICAM-1, and VCAM-1 mRNA expression (panel **A-D**) and protein secretion (panel **E-H**) were measured in HCAEC 7 days after stimulation. Relative quantification (RQ) compared to media-cultured negative control cells were calculated and shown in y-axis. Two-way ANOVA was performed and the statistical differences between direct and indirect stimulation are shown: * $p<0.05$; ** $p<0.01$; *** $p<0.001$; **** $p<0.0001$. Statistical differences between CIT- and/or MAA-modified FIB compared with unmodified FIB are shown: ¶ $p<0.05$; ¶¶ $p<0.01$; ¶¶¶ $p<0.001$; ¶¶¶¶ $p<0.0001$.

Figure S6:

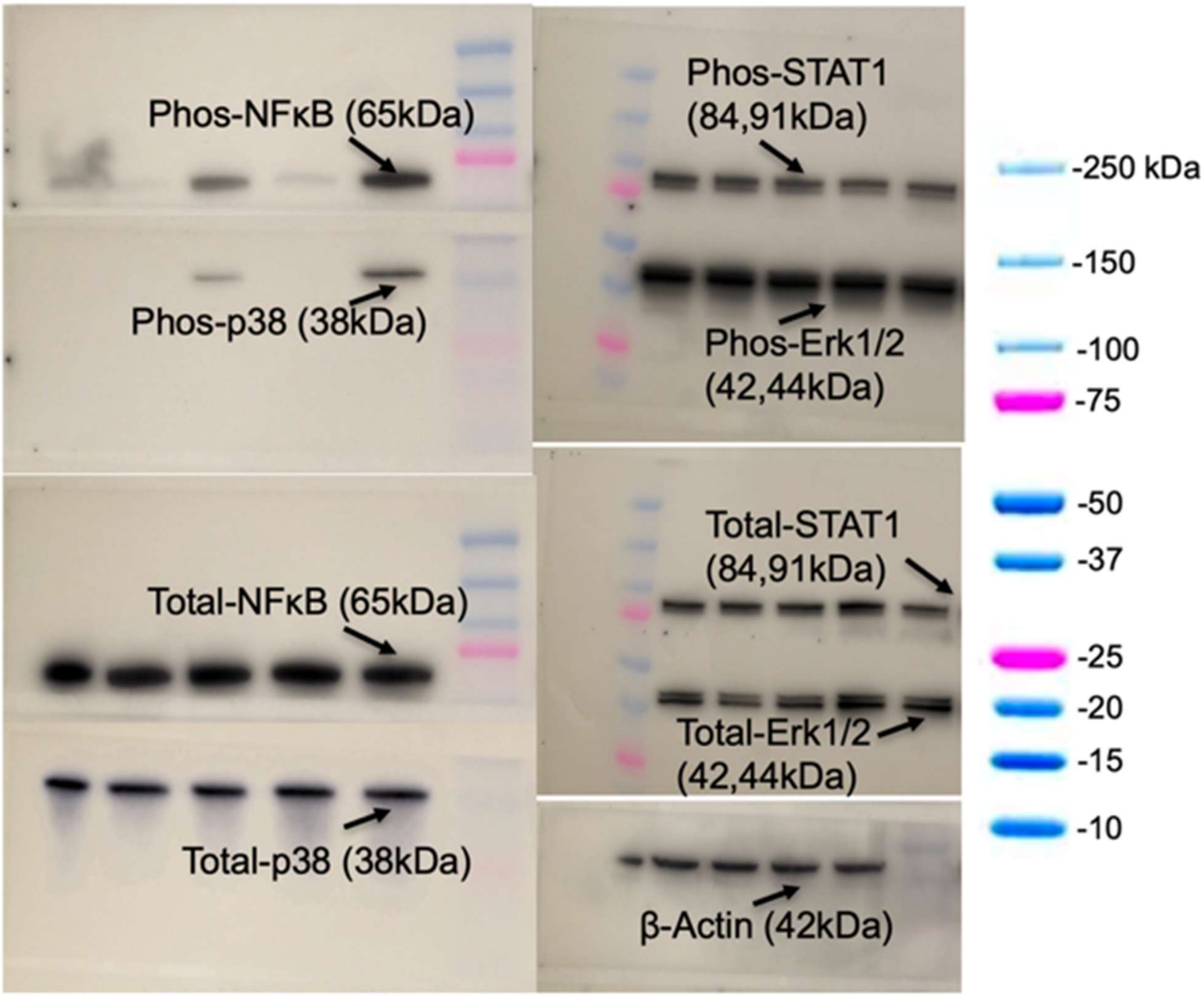

Full image of Western Blot for Figure 5. Bands from left to right are U-937 derived macrophages treated with media, FIB, FIB-CIT, FIB-MAA, and FIB-MAA-CIT.

Figure S7:

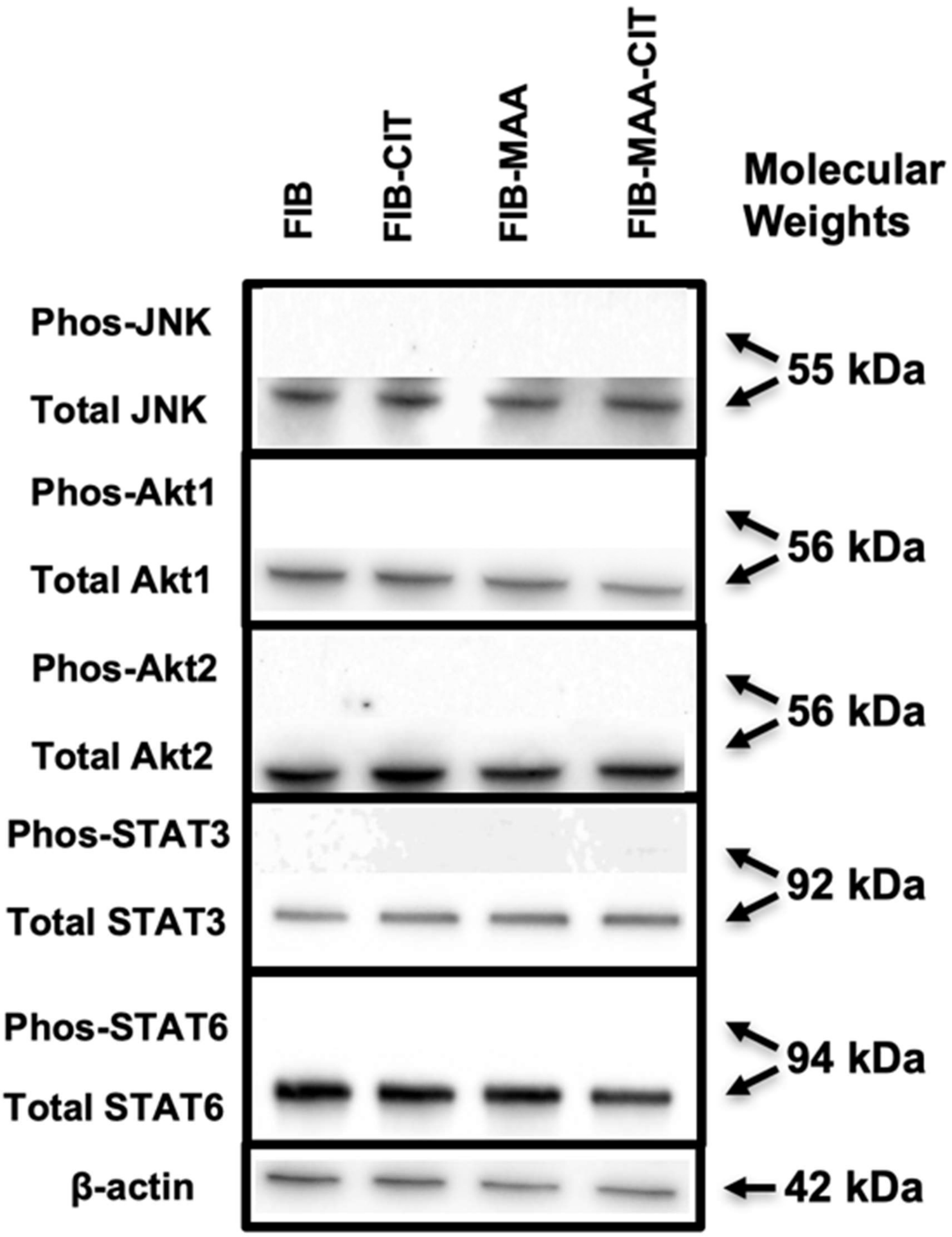

Antigen Stimulated U-937 Derived Macrophages had Undetectable Bands by Western blot for Phosphorylated JNK, Akt1, Akt2, STAT3, and STAT6. Representative images of phosphorylated and total JNK, Akt1, Akt2, STAT3, and STAT6 were shown, with β-actin as loading control.

Figure S8:

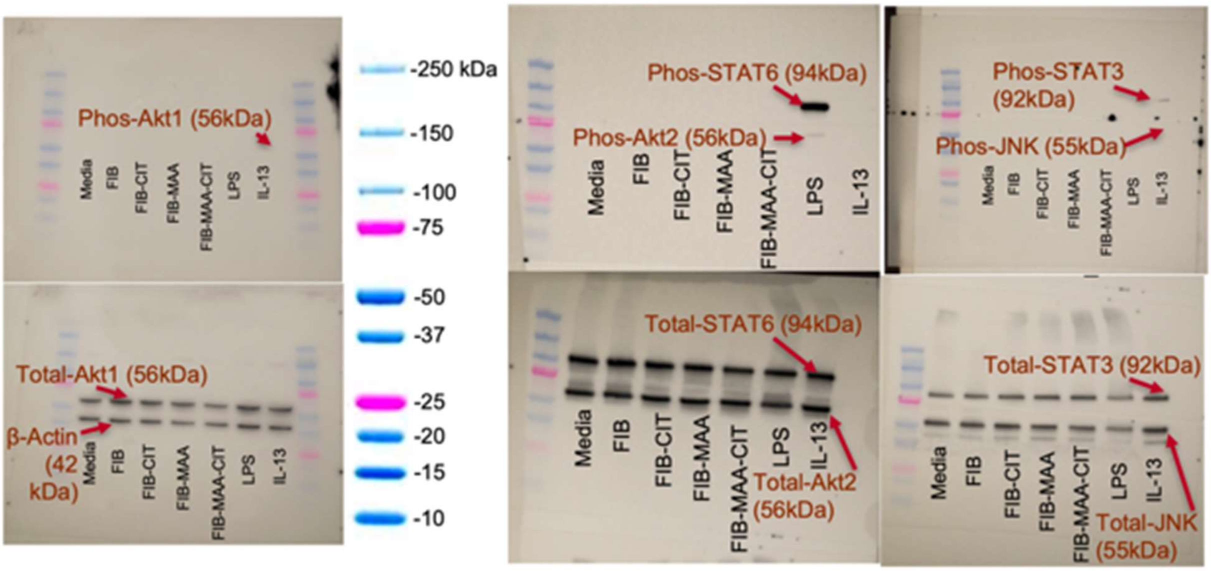

Full image of Western Blot for Figure S7.

Figure S9:

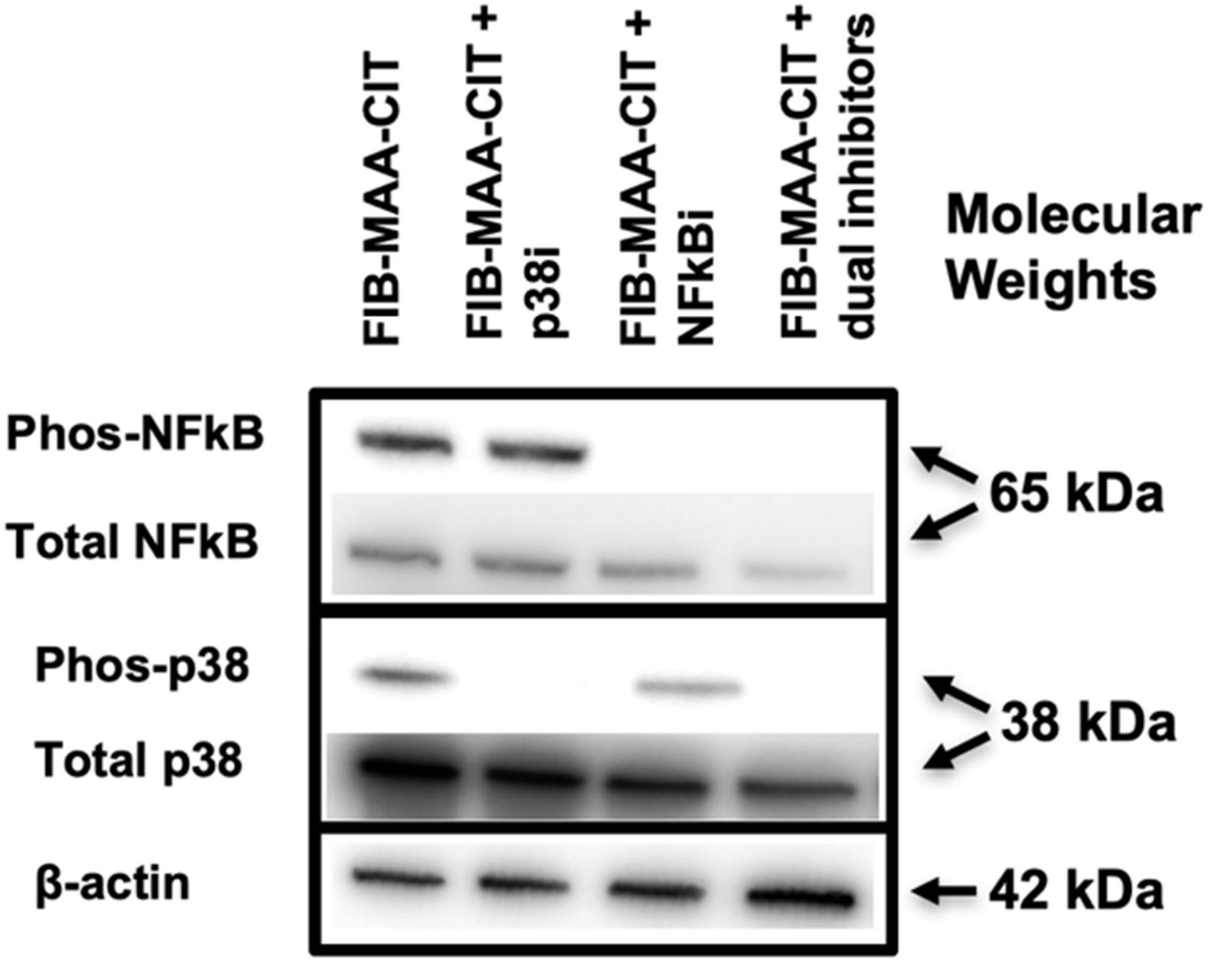

Western Blot Confirming Knock Down of p38 and NF-κB Pathways in U-937 Derived Macrophage following Incubation with Respective Inhibitors. Representative images of phosphorylated and total NF-κB and p38 are shown.

Figure S10:

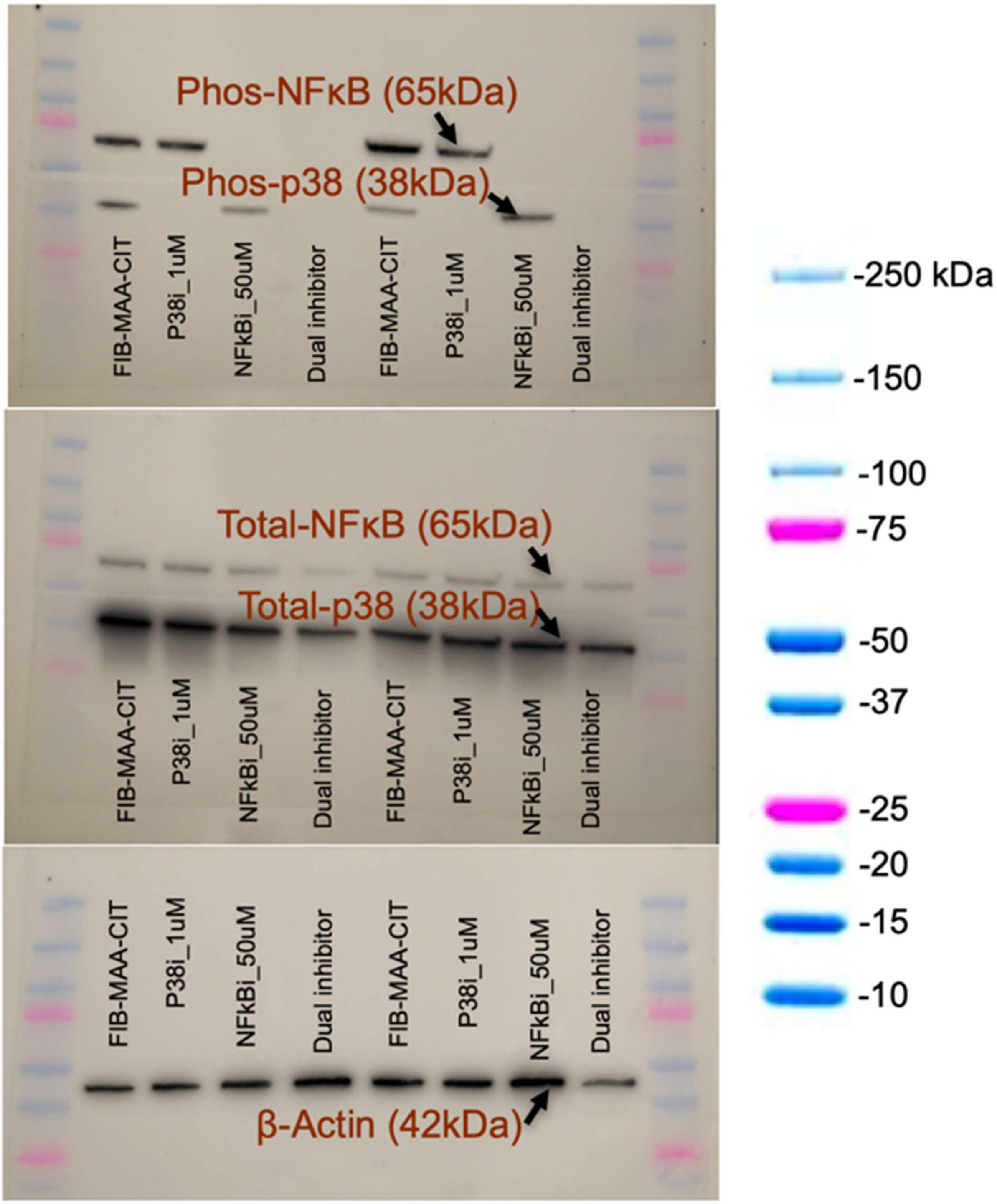

Full image of Western Blot for Figure S9.

Table S1:

Disease Status	Age (years), Sex	Ejection Fraction	Procedure
RA-HF	61, Female	30-35%	Transplant
RA-HF	63, Female	25%	LVAD
RA-HF	56, Female	10%	Transplant
HF	61, Female	5-10%	Transplant
HF	63, Female	10%	LVAD
HF	56, Female	10%	LVAD

Patient Characteristics of Cardiac Tissue Donors. RA = Rheumatoid Arthritis; HF = Heart Failure; LVAD = Left Ventricular Assist Device. Comprehensive disease characteristics are not available.

Table S2:

Disease Status	Age (years), Sex
Healthy Control	27, Male
Healthy Control	25, Male
Healthy Control	27, Female
Healthy Control	26, Female
Healthy Control	28, Female

Patient Characteristics of PBMC Donors.

Major Resources Table

In order to allow validation and replication of experiments, all essential research materials listed in the Methods should be included in the Major Resources Table below. Author are encouraged to use public repositories for protocols, data, code, and other materials and provide persistent identifier and/or links to repositories when available. Authors may add or delete rows as needed.

Antibodies

Targeting Antigen	Vendor	Catalog #	Working Concentration	RRID
Rabbit anti-p38 MAPK	Cell Signaling Technology	9212S	1:1000	AB_330713
Rabbit anti-phospho-p38 MAPK	Cell Signaling Technology	9211S	1:1000	AB_2139682
Rabbit anti-p65 (NFkB)	Cell Signaling Technology	8242S	1:1000	AB_10859369
Rabbit anti-phospho-p65 (NFkB)	Cell Signaling Technology	3033S	1:1000	AB_331284
Rabbit anti-Akt2	Cell Signaling Technology	3063S	1:1000	AB_2225186
Rabbit anti-phospho-Akt2	Cell Signaling Technology	8599S	1:1000	AB_2630347
Rabbit anti-STAT1	Cell Signaling Technology	9172S	1:1000	AB_2198300
Rabbit anti-phospho-STAT1	Cell Signaling Technology	9167S	1:1000	AB_561284
Rabbit anti-Akt1	Cell Signaling Technology	2938S	1:1000	AB_915788
Rabbit anti-phospho-Akt1	Cell Signaling Technology	9018S	1:1000	AB_2629283
Rabbit anti-Erk1/2	Cell Signaling Technology	9102S	1:1000	AB_330744
Rabbit anti-phospho-Erk1/2	Cell Signaling Technology	9101S	1:1000	AB_331646
Rabbit anti-SAPK/JNK	Cell Signaling Technology	9252S	1:1000	AB_2250373
Rabbit anti-phospho-SAPK/JNK	Cell Signaling Technology	9251S	1:1000	AB_331659
Rabbit anti-STAT3	Cell Signaling Technology	12640S	1:1000	AB_2629499
Rabbit anti-phospho-STAT3	Cell Signaling Technology	9145S	1:1000	AB_2491009
Rabbit anti-STAT6	Cell Signaling Technology	5397S	1:1000	AB_11220421

Rabbit anti-phospho-STAT6	Cell Signaling Technology	56554S	1:1000	AB_2799514
Rabbit anti β-Actin	Cell Signaling Technology	4967L	1:1000	AB_330288
HRP Goat anti-Rabbit IgG	Jackson Immuno Research	111-055-046	1:10,000	AB_2338506
Mouse anti-CIT F95 IgMk	Millipore	MABN328	1:100	AB_2938608
A488 Donkey Anti-Mouse IgM	Jackson Immuno Research	715-545-140	1:1000	AB_2340845
Rabbit anti-MAA IgG	Generated and Validated in lab	NA	1:100	NA
Zenon 594 Rabbit IgG Labeling	Invitrogen	Z25307	1:1000	AB_2736956
A488 Mouse IgMk Isotype Control	Biolegend	401617	1:100	AB_493552
A647 Rabbit IgG Isotype Control	Bioss	bs-0295P-A647	1:100	AB_3068583

Elisa

Targeting Antigen	Vendor	Catalog #
MCP-1	Biolegend	438804
IL-6	Biolegend	430515
IL-1β	Biolegend	437015
TNF	Biolegend	430215
ICAM-1	Biolegend	479704
VCAM-1	R&D Systems	DY809

Cultured Cells

Name	Vendor	Race, Age, Sex	Catalog #
U937	ATCC	White, 37, male	CRL-1593.2
HCAEC	Cell Application	Hispanic, 19, male	300k-05a

*See Table S2 above for PBMC donor information.

Others

Name	Vendor	Catalog #
Human Fibrinogen	Cayman	16088
Malondialdehyde	Aldrich Chemical	63287

Acetaldehyde	Aldrich Chemical	402788
Spectrum™ Dialysis Membrane	Thermo Fisher	08-670C
Rabbit Skeletal PAD-4	Cayman	10500
Soybean Trypsin Inhibitor	Thermo Fisher	17075029
Limulus Amebocyte Lysate Assay	Lonza	50-650U
10X Citrate Buffer	Sigma Aldrich	C9999
DAPI	Invitrogen	D1306
Fluoromount-G	Southern Biotech	0100-01
RPMI	Thermo Fisher	11875093
phorbol 12-myristate 13-acetate	Sigma Aldrich	P8139
Fetal Bovine Serum	Sigma Aldrich	F4135
L-Glutamine	Sigma Aldrich	G7513
Penicillin-streptomycin	Sigma Aldrich	P4333
2-mercaptoethanol	Sigma Aldrich	M6250
M-CSF	PeproTech	300-25
RIPA Buffer	Thermo Fisher	89900
Protease Inhibitor Cocktail	Sigma Aldrich	11697498001
Phenylmethylsulfonyl fluoride (PMSF)	Sigma Aldrich	10837091001
BCA Assay	Thermo Fisher	23225
Precision Plus Protein Dual Color Standards	BIO-RAD	1610374
Casein	Sigma Aldrich	70955
BIRB-796	Cayman	10640
BAY-11-7085	Cayman	14795
Meso Endo Growth Medium	Cell Application	212-500
Rneasy Mini Kit	Qiagen	74104
nCounter® CVD Pathophysiology Panel	NanoString	XT-HSCVD-12
High-Capacity RNA-to-cDNA Kit	Applied Biosystems	665544
TaqMan Gene Expression Mix	Applied Biosystems	4369016
CCL2 TaqMan Assays	Thermo Fisher	Hs00234140_m1
IL6 TaqMan Assays	Thermo Fisher	Hs00174131_m1
ICAM1 TaqMan Assays	Thermo Fisher	Hs00164932_m1
VCAM1 TaqMan Assays	Thermo Fisher	Hs01003372_m1
GAPDH TaqMan Assays	Thermo Fisher	Hs02786624_g1
